# Stability of *Cacopsylla pyricola* (Hemiptera: Psyllidae) Populations in Pacific Northwest Pear Orchards Managed with Long-Term Mating Disruption for *Cydia pomonella* (Lepidoptera: Tortricidae)

**DOI:** 10.3390/insects8040105

**Published:** 2017-09-30

**Authors:** Kaushalya G. Amarasekare, Peter W. Shearer

**Affiliations:** 1 Department of Agricultural and Environmental Sciences, College of Agriculture, Tennessee State University, 3500 John A. Merritt Blvd., Nashville, TN 37209, USA; 2 Tree Fruit Research and Extension Center, Washington State University, 1100 N. Western Ave., Wenatchee, WA 98801, USA; peter.shearer@wsu.edu

**Keywords:** pear psylla, codling moth, natural enemies, pear pest insects, mating disruption, biological control

## Abstract

This study focused on conservation biological control of pear psylla, *Cacopsylla pyricola*, in the Pacific Northwest, USA. We hypothesized that insecticides applied against the primary insect pest, codling moth *Cydia pomonella*, negatively impact natural enemies of pear psylla, thus causing outbreaks of this secondary pest. Hence, the objective of this study was to understand how codling moth management influences the abundance of pear psylla and its natural enemy complex in pear orchards managed under long-term codling moth mating disruption programs. We conducted this study within a pear orchard that had previously been under seasonal mating disruption for codling moth for eight years. We replicated two treatments, “natural enemy disrupt” (application of two combination sprays of spinetoram plus chlorantraniliprole timed against first-generation codling moth) and “natural enemy non-disrupt” four times in the orchard. Field sampling of psylla and natural enemies (i.e., lacewings, coccinellids, spiders, *Campylomma verbasci*, syrphid flies, earwigs) revealed that pear psylla populations remained well below treatment thresholds all season despite the reduced abundance of key pear psylla natural enemies in the natural enemy disrupt plots compared with the non-disrupt treatment. We speculate that pear psylla are difficult to disrupt when pear orchards are under long-term codling moth disruption.

## 1. Introduction

Pear psylla, *Cacopsylla pyricola* (Foerster) (Hemiptera: Psyllidae), is one of the most important insect pests of pears (*Pyrus* sp. (Rosaceae)) in Washington, Oregon and other pear-growing states in the USA. If not properly managed, pear psylla can rapidly build up and damage both fruits and trees [1]. Outbreak levels of pear psylla often result in downgraded fruit and/or increased harvest and postharvest costs of pears. Pear psylla is considered as a current and chronic issue in pear crop production and its management is a high priority in these orchard systems [2].

Codling moth, *Cydia pomonella* (Linnaeus) (Lepidoptera: Tortricidae) is another key insect pest of pears. Most pear pest management programs in the USA rely heavily on multiple, targeted insecticide applications for codling moth [3]. In the absence of suitable natural enemies, the use of insecticides is the most common option for codling moth control, although some growers also use mating disruption pheromones for codling moth management [2,4,5]. In contrast, pear psylla has many natural enemies including generalist predators (e.g., *Deraeocoris brevis* (Uhler) (Hemiptera: Miridae), spiders, *Orius* sp. (Hemiptera: Anthocoridae), *Campylomma verbasci* (Meyer) (Hemiptera: Miridae), green lacewings (Neuroptera: Chrysopidae), brown lacewings (Neuroptera: Hemerobiidae) and coccinellids (Coleoptera: Coccinellidae)) and a specialist parasitoid *Trechnites insidiosus* (Crawford) (Hymenoptera: Encyrtidae), although pear psylla are mainly controlled by insecticides [6]. Insecticides used to control codling moth can disrupt biological control of pear psylla by reducing natural enemy abundance in pear orchards [5,7]. 

Pear integrated pest management (IPM) programs that utilize biological control have been investigated [8,9] in the past but not implemented for various reasons including lack of selective insecticides [10,11] and concerns of costly crop damage if insecticide applications are withheld. Our recent experience with pear IPM in Hood River Valley, Oregon, has demonstrated that pear growers who implement pest management programs based on integrated codling moth management with pheromone mating disruption for *C. pomonella* can reduce the need for post bloom insecticide applications for codling moth. This allows pear psylla to be managed with biological control during the summer growing season [2,5,12].

Oregon and Washington pear growers that use pheromone mating disruption for codling moth control are more likely to implement biological control practices in their orchards [11]. However, monitoring for natural enemies often requires a large amount of work to estimate levels of abundance [13]. Fortunately, monitoring natural enemy presence in orchards has recently been enhanced with the development of traps baited with herbivore-induced plant volatiles (HIPV) which attract natural enemies who react to these volatiles like they would their insect prey [14,15,16]. These traps are useful for measuring presence and abundance of adult natural enemies that fly away when being sampled with beat-trays, the most common sampling method used in tree-fruits [14,15,16]. Incorporating other tactics for pear IPM, such as codling moth mating disruption, slows down the development of insecticide resistance and allows biological control to aid pest management.

Our findings from previous studies conducted in two pear orchards that had been under mating disruption for codling moth management for several years or more demonstrated our inability to induce outbreaks of pear psylla following the application of insecticides that were known to be disruptive to natural enemies [5]. We hypothesized that managing codling moth with mating disruption instead of insecticides allows natural enemies to regulate pear psylla populations below treatment threshold. Hence, our objective of this study was to determine the stability of pear psylla populations in a large pear orchard that had been under long-term mating disruption for codling moth. To assess our objective, we utilized plant volatile sticky traps to monitor key natural enemies in addition to conventional monitoring methods such as beat-trays, trunk banding and leaf sampling. The abundance of pear psylla and its natural enemies when natural enemies are disrupted and non-disrupted in a pear orchard under long-term codling moth mating disruption is discussed.

## 2. Materials and Methods

### 2.1. Field Experiments

We conducted this study in a 21 ha pear orchard in White Salmon, Washington, from April to August 2013. The orchard was under a long-term, orchard-wide codling moth mating disruption program since 2005. We selected four blocks, each approximately 1.7 ha of green Bartlett pears from different locations within the orchard and subdivided each block into two plots for the two treatments, i.e., natural enemy disrupt and natural enemy non-disrupt (a control treatment). A typical regional early season spray program which targeted overwintering pear psylla was uniformly applied across the study site at 935 L/ha water for each application. Mineral oil (Omni Supreme Spray^®^, Helena Chemical Company, Collierville, TN, USA) (37.4 L/ha) was applied 24 February, mineral oil (18.7 L/ha) + pyriproxyfen (Esteem^®^ 35%, Valent U.S.A., Walnut Creek, CA, USA) (350 g/ha) on 8 March, followed by pyridaben ((Nexter^®^ 75%, Gowan Co. Yuma, AZ, USA) (560.5 g/ha) plus mancozeb (Penncozeb^®^ 75%, United Phosporus, Inc., King of Prussia, PA, USA) (8.96 kg/ha) on 1 April, mancozeb (75%) 8.96 kg/ha) plus kaolin (Surround^®^ 95%, Tessenderlo Kerley, Inc., Phoenix, AZ, USA) (56 kg/ha) on 21 April, and finally abamectin (Zoro^®^ 1.9%, Cheminova, Inc., Research Triangle Park, NC, USA) (1.46 L/ha) plus mineral oil (98%) (9.3 L/ha) which was applied about 10 days after petal fall.

We applied our natural enemy disrupt treatment consisting of a mixture of chlorantraniliprole (Altacor^®^ 35%, DuPont Crop Protection, Wilmington, DE, USA) (280 g/ha), spinetoram (Delegate^®^ 25%, Dow Agro Sciences LLC, Indianapolis, IN, USA) (490 g/ha) plus an adjuvant (mineral oil) (4.7 L/ha) at the rate of 935 L water/ha to the plots on 25 May and 12 June to disrupt natural enemies in the natural enemy disrupt treatment. Chlorantraniliprole and spinetoram were included because of their detrimental impacts to green lacewings [17,18] while spinetoram has also been shown to have negative population effects on green lacewings and *D. brevis* [17,18,19] and other natural enemies [20]. These applications were timed to target newly hatched codling moth larvae. We then assessed the effects of insecticide disruption on the abundance of pear psylla and its natural enemies and then compared that with arthropod abundance in the non-disrupted blocks. To avoid the edge effect of the other treatment within a block, we conducted all sampling for monitoring of pear psylla and natural enemies in the center of each treatment plot.

### 2.2. Pear Psylla Monitoring

Pear psylla eggs and nymphs were monitored weekly from fruit spur leaf samples collected from 6 May to 20 May and then from shoot leaf samples until harvest. We handpicked leaves from spurs from five pear trees per treatment per block at the rate of ten fruit spur leaves per tree and then checked them under the microscope for pear psylla eggs and nymphs and counted the numbers present. After 20 May, we sampled ten pear trees per treatment per block weekly at the rate of one shoot per tree for monitoring pear psylla eggs and nymphs. We removed each shoot from the upper canopy of the tree using a telescoping pole pruner. We placed the shoots collected from each treatment block in a brown paper bag and put the bag in a cooler with icepacks before transporting them to the laboratory. At the laboratory, we randomly selected three leaves per shoot (one fully expanded distal, medial and proximal leaf per shoot) and checked the lower and upper epidermis of each leaf under the microscope for pear psylla eggs and nymphs.

Once a week, we sampled fifteen pear trees per treatment per block using a beat-tray [21] at the rate of one tray per tree for pear psylla adult monitoring. For each beat-tray sample, we chose a horizontal limb approximately 1.5 m above from the ground from the selected tree and counted the adult pear psylla that fell onto the tray after three limb-jarring taps.

### 2.3. Natural Enemy Monitoring

#### 2.3.1. Beat-Trays

We used the beat-tray samples collected for pear psylla abundance to monitor the presence of different categories of immature and adult natural enemies. We aspirated some adult natural enemies into vials with alcohol for later identification. We aspirated lacewing and coccinellid larva individually into plastic vials and reared them for identification. For these, we brought the immature insects to the laboratory and fed them once a week with *Ephestia kuehniella* Zeller eggs (Lepidoptera: Pyralidae) (Beneficial Insectary, Redding, CA, USA) until adult emergence and then identified them to family, genus and/or species and numbers emerged were added to the beat-tray data.

#### 2.3.2. Tree-Banding

We conducted bi-weekly tree-banding from 6 May to 20 Aug. We placed 3.8-cm-width cardboard bands (Model S-11450, ULINE, Chicago, IL, USA) around tree trunks at the rate of one band per tree for a total of 20 bands for each treatment per block. We selected the trees from the middle section of each treatment plot within a block and placed cardboard bands around each tree trunk approximately 45 cm from the ground and stapled the band to the trunk. After 14 days, we collected the bands and replaced them with a new set of 20 bands. We placed the bands collected from each treatment block together in a large plastic bag placed within in a cooler with icepacks before transporting them to the laboratory. At the laboratory, we checked the collected bands for earwig nymphs and adults (counted), immature and adult spiders (counted and preserved in 75% alcohol for identification), larvae and pupae of lacewings (counted and reared) and larvae and pupae of coccinellids (counted and reared). We reared the collected larvae and pupae and identified the emerged adults as mentioned above and numbers emerged were added to the tree-banding data. Collected spiders were sent to the USDA-ARS (United State Department of Agriculture-Agricultural Research Service), Yakima Agricultural Research Laboratory, Wapato, WA, for identification.

#### 2.3.3. HIPV and Pheromone Traps

We deployed three HIPV and one sex pheromone lure traps with either a white or yellow sticky card (Suterra LLC, Bend, OR, USA) in each treatment plot on 30 April (Lure 1: Squalene (Sigma-Aldrich, St. Louis, MO, USA): for the green lacewing *Chrysopa nigricornis* Burmeister (Neuroptera: Chrysopidae) (white sticky card), Lure 2: separate lures of acetic acid, methyl salicylate and 2-phenylethanol (Sigma-Aldrich, St. Louis, MO, USA) hung together for green lacewings *Chrysoperla* sp. (Neuroptera: Chrysopidae) (white sticky card), Lure 3: a mixture of 2-phenylethanol and geraniol (Sigma-Aldrich, St. Louis, MO, USA) for Syrphids (yellow sticky card) and Lure 4: *Campylomma* lure (Mullein bug (*Campylomma verbasci* (Meyer)) sex pheromone flexlure (Contech Enterprises Inc., Victoria, BC, Canada) for *Campylomma verbasci* (Hemiptera: Miridae) (yellow sticky card)) (Table 1)).

We prepared lure bags (5 cm × 5 cm) for each HIPV trap by heat sealing different thicknesses of polyvinyl tubing (Associated Bag Company, Milwaukee, WI, USA) (Table 1). We selected the gauge of tubing by considering each lure chemical type (Table 1) from previously conducted studies [14,15,16]. We pipetted each lure chemical at the rate mentioned in the Table 1 onto a cotton wick (Patterson Dental, St. Paul, MN, USA) which was placed inside each lure bag and then immediately heat-sealed the bag. We stored the prepared lure bags in a freezer until use. We prepared each trap by attaching a lure and a sticky card (white or yellow, Table 1) to a wire trap hanger (Suterra USA, Bend, OR, USA) and then secured the lure with a small binder clip to prevent the lure getting stuck to the sticky card. We hung each trap in a tree branch that was approximately 1.5 m above from the ground. We collected and replaced the traps weekly. We wrapped the sticky side of each collected card with plastic wrap immediately after its removal and placed all collected sticky cards in a cooler with icepacks before transporting them to the laboratory. At the laboratory, we placed the collected sticky cards in a freezer till they were checked under a microscope to identify different categories of natural enemies. Once a month, we removed and replaced lures with new ones.

### 2.4. Data Analysis

We used a randomized complete block design (RCBD) in the analysis. Generalized linear models with sampling date as a repeated measure (SAS PROC MIXED Statistical Analysis Software Proc Mixed Procedure) were used to analyze the effects of treatment and sampling date for both interaction and main effects. When there was a significant interaction (significance at *p* ≤ 0.05) between fixed main effects, the effect of treatment was tested separately for each sampling date. In the absence of an interaction, the main effects were tested for significance (significance at *p* ≤ 0.05). Treatment comparisons within a given sampling date were analyzed using t-test and all significant differences are noted on graphs using an asterisk (* = significance at *p* ≤ 0.05). We carried out all statistical procedures using SAS (Statistical Analysis Software) [22].

## 3. Results

### 3.1. Pear Psylla Monitoring

**Figure 1:**
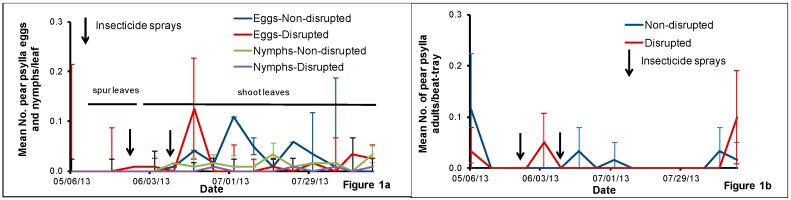
Mean number (±SE) of pear psylla eggs and nymphs per spur or shoot leaf (**a**) and pear psylla adults per beat-tray (**b**) for the samples collected in the natural enemy non-disrupted and disrupted plots in a pear orchard in White Salmon, Washington, USA, from May to August 2013. Significant differences between the treatments when present are noted on the graph using an asterisk (* = significance at *p* ≤ 0.05).

We did not find any pear psylla eggs and nymphs on sampled spur leaves in either treatment. For the abundance of pear psylla eggs on shoot leaves, the interaction between treatment and sampling date (*F* = 1.16, *df* = 12, 75, *p* = 0.33) and the main effects treatment (*F* = 0.48, *df* = 1, 75, *p* = 0.49) and sampling date (*F* = 1.17, *df* = 12, 75, *p* = 0.32) were not significant. Low numbers of pear psylla eggs and nymphs were detected on shoot leaves collected from the two treatments (Figure 1a). For pear psylla nymphs on shoot leaves, the interaction between treatment and sampling date (*F* = 0.50, *df* = 12, 75, *p* = 0.91) and the main effect sampling date (*F* = 0.80, *df* = 12, 75, *p* = 0.65) were not significant although the main effect treatment (*F* = 6.40, *df* = 1, 75, *p* = 0.01) was significant. The treatment effect was significant when analyzed with pooled sampling date (*F* = 6.28, *df* = 1, 102, *p* = 0.01) indicating that there were more pear psylla nymphs detected in the non-disrupted treatment than in the disrupted treatment. Overall, pear psylla nymph counts were well below treatment threshold levels (0.3 nymphs per leaf [10] throughout the season. Very few adult pear psylla were detected in beat-tray samples in both non-disrupted and disrupted plots throughout the study period (Figure 1b). For the abundance of pear psylla adults in beat-tray samples, the interaction between treatment and sampling date (*F* = 1.39, *df* = 15, 93, *p* = 0.17) and the main effect treatment (*F* = 0.08, *df* = 1, 93, *p* = 0.78) were not significant although the main effect sampling date (*F* = 2.30, *df* = 15, 93, *p* = 0.01) was significant. The sampling date effect was significant when analyzed with pooled treatment (*F* = 2.2, *df* = 15, 112, *p* = 0.01) indicating the presence of more adult psylla in some of the sampling dates than in the others irrespective of the treatment.

### 3.2. Natural Enemy Monitoring

#### 3.2.1. Beat-Trays

The categories of natural enemies sampled were green and brown lacewings adults (collected and preserved in 75% ethyl alcohol) and larvae (reared), coccinellid adults (collected live and identified and released) and larvae (reared), spiders (collected and preserved in 75% ethyl alcohol), and earwigs (counted and released). All natural enemies reared to adults or collected as adults were identified to the family, genus and/or species and the counts were added to the beat-tray data.

**Figure 2:**
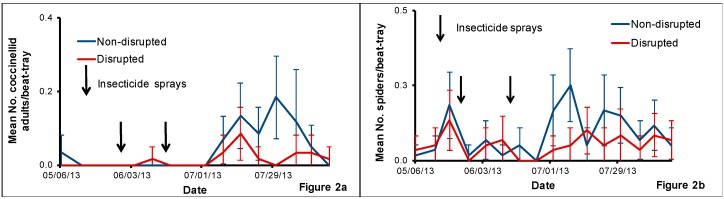
Mean number (±SE) of coccinellid adults (**a**) and spiders (**b**) per beat-tray collected in the natural enemy non-disrupted and disrupted plots in a pear orchard in White Salmon, Washington, USA, from May to August 2013. Significant differences between the treatments when present are noted on graphs using an asterisk (* = significance at *p* ≤ 0.05).

We observed a low number of coccinellid adults on beat-tray samples after the two insecticide sprays in May and June (Figure 2a). The number of coccinellids collected increased in July and then gradually declined in August. For the abundance of coccinellid adults in beat-tray samples, the interaction between treatment and sampling date (*F* = 1.41, *df* = 15, 93, *p* = 0.16) was not significant while the main effect of treatment (*F* = 6.41, *df* = 1, 93, *p* = 0.01) and sampling date (*F* = 2.95, *df* = 15, 93, *p* = 0.001) were significant. When we analyzed the treatment effect for each sampling date, there were more coccinellids observed in the non-disrupted block than on the disrupted block on three sampling dates in late July and early August (23 July: *t* = 1.56, *df* = 1, 93, *p* = 0.01; 30 July: *t* = 4.29, *df* = 1, 93, *p* < 0.0001; 7 August: *t* = 1.95, *df* = 1, 93, *p* = 0.04). Approximately 90% of the coccinellids in beat-tray samples were *Hippodamia convergens* Guérin-Méneville (and ~10% were *Cycloneda munda* (Say, 1835). All adults emerged from collected coccinellid larvae and pupae were *H. convergens*.

We observed spiders in all beat-tray samples collected from both non-disrupted and disrupted plots throughout the sampling period, although the numbers were low after the insecticide sprays and until early July (Figure 2b). For the mean number of spiders in beat-tray samples, the interaction between treatment and sampling date (*F* = 2.17, *df* = 15, 93, *p* = 0.01) and main effects treatment (*F* = 9.57, *df* = 1, 93, *p* = 0.003) and sampling date (*F* = 4.87, *df* = 15, 93, *p* < 0.0001) were significant. When we analyzed the treatment effect for each sampling date, we found a significantly higher number of spiders in the beat-tray samples collected from the non-disrupted treatment than in the disrupted treatment in three weeks of July (2 July: *t* = 3.00, *df* = 1, 93, *p* = 0.004; 9 July: *t* = 4.13, *df* = 1, 93, *p* < 0.0001; 23 July: *t* = 2.63, *df* = 1, 93, *p* = 0.014). Spiders were the dominant natural enemy found in beat-trays.

We collected a negligible number of green and brown lacewings (adults and larvae), *Trechnites* sp. (adults), *D. brevis* (adults and nymphs), *C. verbasci* (adults), Syrphids (larvae), *Orius* sp. (adults) and earwigs (nymphs and adults) and coccinellid larvae from the beat-tray samples during the monitoring period. All green lacewing adults (collected and released) and larvae (collected and reared) from beat-tray samples were *Chrsyoperla* sp. 

#### 3.2.2. Tree-Banding

**Figure 3:**
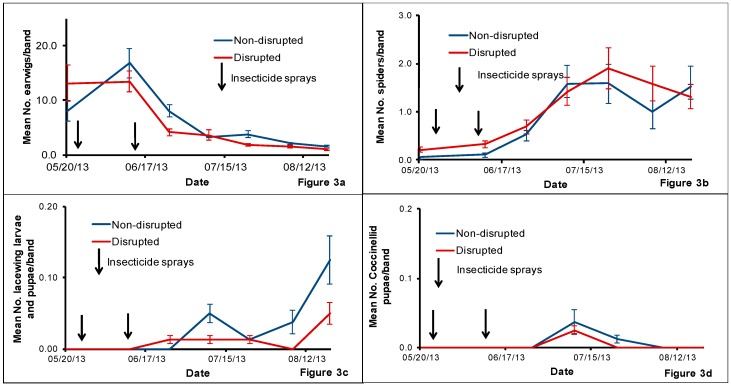
Mean number (±SE) of earwigs (**a**), spiders (**b**) lacewing larvae and pupae (**c**) and coccinelid pupae (**d**) per cardboard band collected from tree-banding in the natural enemy non-disrupted and disrupted plots in a pear orchard in White Salmon, Washington, USA, from May to August 2013. Significant differences between the treatments when present are noted on graphs using an asterisk (* = significance at *p* ≤ 0.05).

We collected European earwigs *Forficula auricularia* (L.) (Dermaptera: Forficulidae), from cardboard bands fastened on tree trunks in both non-disrupted and disrupted treatments during the sampling period (Figure 3a). For the abundance of earwigs in cardboard bands, the interaction between treatment and sampling date (*F* = 0.52, *df* = 6, 39, *p* = 0.79) and the main effect treatment (*F* = 0.21, *df* = 1, 39, *p* = 0.65) were not significant although the other main effect sampling date (*F* = 6.28, *df* = 6, 39, *p* = 0.0001) was significant. The sampling date effect was significant when analyzed with pooled treatment (*F* = 5.73, *df* = 6, 49, *p* = 0.0001) indicating the presence of more earwigs in some of the sampling dates than in the others irrespective of the treatment. Earwig numbers declined gradually after the second insecticide spray in mid-June, but this was apparent in both treatments.

Spiders were the next dominant natural enemy collected from tree-banding (Figure 3b). For the abundance of spiders in cardboard bands, the interaction between treatment and sampling date (*F* = 0.15, *df* = 6, 39, *p* = 0.99) and the main effect treatment (*F* = 0.30, *df* = 1, 39, *p* = 0.58) were not significant although the main effect sampling date (*F* = 3.47, *df* = 6, 39, *p* = 0.01) was significant. The sampling date effect was significant when analyzed with pooled treatment (*F* = 2.69, *df* = 6, 49, *p* = 0.02) indicating the presence of more spiders in some of the sampling dates than in the others irrespective of the treatment. We observed a gradual increase of spiders collected from tree-banding in both treatments after the two insecticide sprays (Figure 3b). Overall, we collected 1124 immature and adult spiders and 95% of them were in the family Linyphiidae. The family Linyphiidae consists of very small spiders and it is the second largest family of spiders after the family Salticidae. Out of all collected spiders in the family Linyphiidae, ~37% were identified as *Spirembolus* sp. and we speculate that ~44% of the Linyphiidae spiders were probable *Spirembolus* sp. and the remaining ~13% were unidentified Linyphiidae immatures.

We did not collect any lacewing larvae and pupae from the tree-banding from 20 May to mid-June, the time period between the two insecticides sprays (Figure 3c). There was a gradual increase in the number of lacewing larvae and pupae found in bands collected from both treatments after the second insecticide spray although their abundance was low in both non-disrupted and disrupted plots. For the mean number of lacewing larvae and pupae in cardboard bands, the interaction between treatment and sampling date (*F* = 0.75, *df* = 6, 39, *p* = 0.61) and the main effect treatment (*F* = 2.06, *df* = 1, 39, *p* = 0.16) were not significant although the main effect sampling date (*F* = 2.89, *df* = 6, 39, *p* = 0.02) was significant. The sampling date effect was significant when analyzed with pooled treatment (*F* = 2.77, *df* = 6, 49, *p* = 0.02) indicating the presence of more lacewing larvae and pupae in some of the sampling dates than in the others irrespective of the treatment. We collected only a few coccinellid pupae from cardboard bands throughout the study period (Figure 3d). For the abundance of coccinellid pupae on bands, the interaction between treatment and sampling date (*F* = 0.15, *df* = 6, 39, *p* = 0.99) and the main effects treatment (*F* = 0.35, *df* = 1, 39, *p* = 0.56) and sampling date (*F* = 2.16, *df* = 6, 39, *p* = 0.07) were not significant.

#### 3.2.3. HIVP and Pheromone Traps

**Figure 4:**
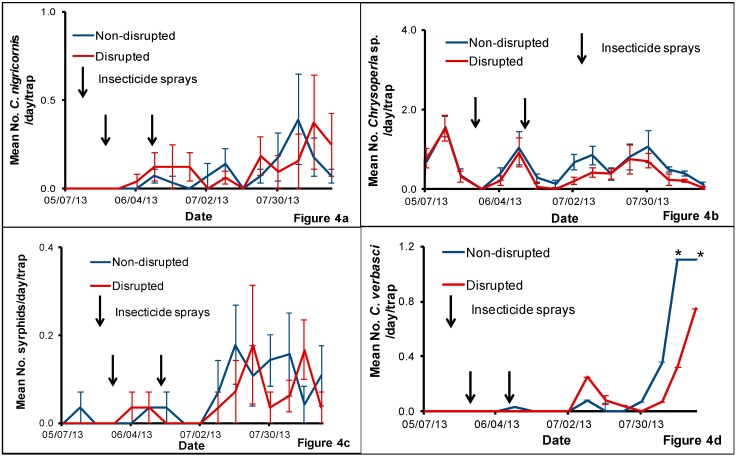
Mean number (±SE) of adult *Chrysopa nigricornis* (**a**), *Chrysoperla* sp. (**b**), syrphids (**c**) and *Campylomma verbasci* (**d**) captured per day per trap on HIPV traps placed in the natural enemy non-disrupted and disrupted plots in a pear orchard in White Salmon, Washington, USA, from May to August 2013. Significant differences between the treatments when present are noted on graphs using an asterisk (* = significance at *p* ≤ 0.05).

The number of the green lacewing *C. nigricornis* caught in weekly HIPV traps was low (Figure 4a). For the abundance of *C. nigricornis* in HIPV traps, the interaction between treatment and sampling date (*F* = 0.69, *df* = 15, 93, *p* = 0.79) and the main effect treatment (*F* = 0.38, *df* = 1, 93 *p* = 0.54) were not significant although the main effect sampling date (*F* = 1.93, *df* = 15, 93, *p* = 0.03) was significant. The sampling date effect was significant when analyzed with pooled treatment (*F* = 1.95, *df* = 15, 112, *p* = 0.02) indicating the presence of more *C. nigricornis* in some of the sampling dates than in the others irrespective of the treatment. We started to capture *C. nigricornis* in both treatments from 4 June, which was a week before the second insecticide spray. Although the number of *C. nigricornis* caught in HIPV traps gradually increased in July and August, overall the populations were low in both treatments.

We captured *Chrysoperla* sp. in both disrupted and non-disrupted treatments throughout the study period (Figure 4b). For the mean number of *Chrysoperla* sp. in HIPV traps, the interaction between treatment and sampling date (*F* = 0.36, *df* = 15, 93, *p* = 0.99) was not significant although the main effect treatment (*F* = 5.48, *df* = 1, 93, *p* = 0.02) and the main effect sampling date (*F* = 9.67, *df* = 15, 93, *p* < 0.0001) were significant. There was no significant difference between the two treatments in respective sampling date although there were significant differences when compared treatments with different sampling dates. There was a decline in *Chrysoperla* sp. populations after the two insecticide sprays in both disrupted and non-disrupted treatments. Although low in numbers, *Chrysoperla* populations were continuously present in both non-disrupted and disrupted plots throughout the trapping period.

We captured a low number of syrphids on HIPV traps (Figure 4c). For the abundance of syrphids in HIPV traps, the interaction between treatment and sampling date (*F* = 0.84, *df* = 15, 93, *p* = 0.63) and the main effect treatment (*F* = 0.87, *df* = 1, 93, *p* = 0.35) were not significant, although the main effect sampling date (*F* = 2.20, *df* = 15, 93, *p* < 0.01) was significant. The sampling date effect was significant when analyzed with pooled treatment (*F* = 2.22, *df* = 15, 112, *p* = 0.001) indicating the presence of more syrphids in some of the sampling dates than in the others irrespective of the treatment. Syrphids were mainly caught during the months of July and August in both disrupted and non-disrupted plots.

Adult *C. verbasci* started appearing on pheromone traps in early July and then the numbers rapidly increased on traps in both non-disrupted and disrupted plots (Figure 4d). For the abundance of *C. verbasci* in HIPV traps, the interaction between treatment and sampling date (*F* = 1.53, *df* = 15, 93, *p* = 0.11) was not significant, although the main effect treatment (*F* = 2.63, *df* = 1, 93 *p* = 0.04) and the main effect sampling date (*F* = 8.85, *df* = 15, 93, *p* < 0.01) were significant. When we analyzed the treatment effect for each sampling date, there were more *C. verbasci* observed in the non-disrupted block than on disrupted block on the last two sampling dates in August (13 August: *t* = 4.19, *df* = 1, 93, *p <* 0.0001; 20 August: *t* = 2.03, *df* = 1, 93, *p* = 0.04).

## 4. Discussion

In a previous study, we were unable to induce outbreaks of pear psylla following insecticide applications that were designed to interfere with conservation biological control of pear psylla [5]. We concluded, in part, that previous reductions in insecticide applications applied for codling moth management in an area-wide codling moth mating disruption program caused these orchards to have several years of low levels of pear psylla, which carried over during the two years we conducted that study. In this current study, we investigated the impacts of a mixture of disruptive codling moth insecticide sprays on natural enemies of pear psylla. We observed a similar trend in this study; that it is difficult to disrupt pear psylla in orchards which are under long-term mating disruption for codling moth.

We applied a mixture of two reduced-risk insecticides i.e., chlorantraniliprole and spinetoram to disrupt the natural enemies in the “natural enemy disrupted” treatment plots in this study. These two insecticides are used for codling moth management in tree fruit orchards. Previous laboratory and field studies on lethal and sublethal effects of reduced-risk insecticides on natural enemies show detrimental impact of chlorantraniliprole to chrysopids [5,8,17]. Spinetoram has also been shown to have negative population effects on *D. brevis* [19] and other natural enemies [5,20].

The pear orchard we used for this study was under codling moth mating disruption for more than eight years. One major result of this practice is that this orchard rarely had insecticides applied against pear psylla during summer growing seasons. Hence, we hypothesized that this would help conserve natural enemies and minimize pear psylla outbreaks. Field sampling of pear psylla and natural enemies revealed that pear psylla populations remained well below treatment thresholds all season despite the reduced abundance of some key pear psylla natural enemies in both the natural enemy disrupted plots compared with the non-disrupted treatment.

The uniqueness of this orchard is that these pest management practices have helped to build up large populations of spiders throughout the orchard. Irrespective of the method of monitoring used, spiders were the most dominant natural enemy detected in this particular orchard over the other natural enemies such as coccinellids, green lacewings, *D. brevis* and *C. verbasci*, etc. that are generally found in pear orchards. Although we cannot point out the exact reasons for the low populations of some of the natural enemies in non-disrupted plots, we speculate that the historical use of codling moth mating disruption conserved natural enemies at a background level that kept pear psylla in check. Subsequently, natural enemy population trends mirrored pear psylla populations where low levels of pear psylla equates to low abundance of natural enemies and/or visa-versa.

Previously, spiders were shown to be more abundant in minimally managed orchards [23]. In our study, we observed that spiders can be very abundant compared with levels of other natural enemies in pear orchards with minimal exposure to insecticides during the summer growing season. The spider counts from beat-trays and tree-banding demonstrate the continuous abundance of spider populations in both non-disrupted and disrupted plots. While we were unable to isolate the effects of spiders on pear psylla populations, it is quite possible that spiders can be a dominant yet often overlooked biological control component in pear orchards and possibly an important regulator of pear psylla populations.

Spiders are one of the most common generalist predators collected in other tree-fruit orchards in the Pacific Northwest. In this study, the majority of the collected spiders belonged to the family Linyphiidae. Studies conducted in apple orchards in two geographic regions (Yakima and Wenatchee) in Central Washington State show that Linyphiidae is one of the common families of spiders found in this region [24]. Our results support the results of the previous studies conducted to find the importance of spiders in controlling arthropod pests in orchard crops in the Pacific Northwest.

Green lacewings are important generalist predators for orchard cropping systems in the USA. They are commonly found in pear orchards in Oregon. *Chrysoperla johnsoni* Henry, Wells and Pupedis is one of the most common green lacewing species found in the western United States, including Washington, Oregon, Idaho, California and Arizona [25,26,27,28]. In this study, we found continuous abundance of *Chrysoperla* sp. throughout the study period although the trap catches were less than two adults per day per trap in both treatments. *Chrysopa nigricornis* is the other important green lacewing species for orchard cropping systems in the Western USA [5]. The HIPV trap catches for *C. nigricornis* were less than 0.5 per day per trap in both treatments. In addition to disruption by insecticides, we speculate that spiders (being generalist predators) may have consumed the green lacewing egg, larval and pupal stages existed within the orchard and hence lowered their population in the experiment plots. We also speculate that spiders may have directly or indirectly contributed to the lower population levels of other natural enemies either by negatively affecting their searching behavior or through predation, thus reducing their presence in the orchard.

Earwigs are important biological indicators to evaluate non-target effects of insecticides in agricultural cropping systems. They are commonly found generalist predators in tree fruit orchards in the USA [29]. European earwigs *F. auricularia* is the most common earwig species found in Pacific Northwest tree fruit orchards [29]. Previous studies show that some of the reduced-risk and organophosphate replacement insecticides such as spinosad (a compound related to spinetoram) and novaluron, respectively, are highly toxic to earwigs [30]. In this study, we collected a large number of European earwigs from the cardboard bands although their numbers declined drastically after the second insecticide spray in both treatments.

Herbivore-induced plant volatiles (HIPVs) are important tools to monitor biodiversity of natural enemies in agricultural cropping systems [14,15,16,31]. In this study, we used three HIPV traps to monitor green lacewings (*Chrysoperla* spp. and *C. nigricornis*) and syrphids. By utilizing these newly available tools [14,15,16,31], we were able to continuously monitor the targeted natural enemies throughout the study period. Since we were targeting for natural enemies with active flight in adult stage, the use of beat-tray sampling, the other common method used in tree-fruit orchard, was not an effective option. Hence, the availability of these HIPV monitoring tools has helped us in monitoring the natural enemies efficiently and effectively in this study. These tools will be very useful for studies on evaluating natural enemies in agricultural cropping systems as well as monitoring adverse effects of insecticides on natural enemies.

While this and previous studies [5,29] demonstrated that applications of insecticides classified as disruptive do not always cause pest outbreaks, the common factor in our pear studies is that the study sites had been under long-term codling moth mating disruption. The information we collected in this study demonstrates that long-term mating disruption for codling moth control can most likely lead to reductions in pesticide use, increased abundance of natural enemies and reductions in secondary pest outbreaks. This ultimately reduces costs of pear IPM programs, which has already been observed locally [12]. This information will benefit pest managers in their IPM programs. These results could be well utilized to investigate future studies on the benefits of long-term codling moth mating disruption programs as well as the importance of conservation biological control for arthropod pest management in orchard cropping systems. This study also suggests that biological control of pear psylla is possible during summer months.

## Figures and Tables

**Table 1:** Target natural enemy and trap information for herbivore-induced plant volatiles (HIPVs) and a pheromone lure used to monitor natural enemy abundance in a pear orchard in White Salmon, WA, during May to August 2013.

Target Natural Enemy	Lure (HIPV/Pheromone)	Amount (mL)	Bag Thickness (mil)	Cotton Wick Size	Sticky Card
*Chrysopa nigricornis*	Squalene	1	6	small	white
*Chrysoperla* sp.	Acetic acid + Methyl salicylate + 2-phenylethanol (lures in separate bags and hung together as a single trap)	3	4	large	white
		3.5	6	large	-
		1	1.5	small	-
Syrphids	2-phenylethanol + Geraniol (lures in a single bag)	1	2.0	medium	yellow
		2	-	-	-
*Campylomma* sp.	*Campylomma* lure (sex pheromone flexlure)	-	-	-	yellow
